# Editorial: Nano-Imaging in Translational Cancer Medicine

**DOI:** 10.3389/fbioe.2022.969430

**Published:** 2022-07-22

**Authors:** Fred C. Lam

**Affiliations:** ^1^ Division of Neurosurgery, Saint Elizabeth’s Medical Center, Brighton, MA, United States; ^2^ Koch Institute for Integrative Cancer Research at MIT, Cambridge, MA, United States

**Keywords:** nanotechnology, cancer therapeutic, diagnostic imaging, theranostic, fluorescence guided surgery

The use of nanomaterials has become more prevalent in modern day society since that fateful day at the California Institute of Technology in 1959 when American physicist and Nobel laureate Richard Feynman envisioned the ability to construct machines down to the molecular level during his lecture “There’s Plenty of Room at the Bottom” (Feynman, 1960). Fast forward 30 years, the discovery of carbon nanotubes (CNTs) by Iijima (1991), led to widespread applications of CNTs ranging from carbon fibers to improve the mechanical and thermal stability of polymers used in machine coatings, boat hulls, and water filters, to rechargeable batteries and thin-film electronics (De Volder et al., 2013). The emerging introduction of CNTs and other nanomaterials in biomedical applications continues to push the envelope of what is possible for improving the future of human health and well-being (reviewed in Kinnear et al., 2017) (Kinnear et al., 2017).

**FIGURE 1:**
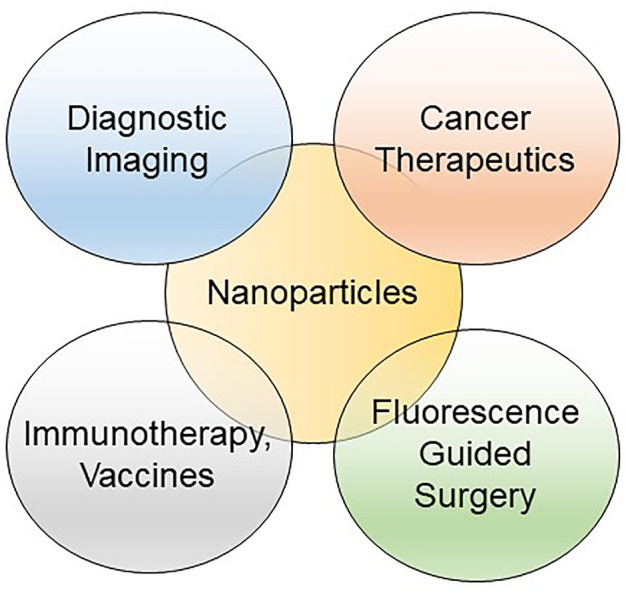
Venn diagram of the applications for nanoparticles in translational oncology.

In this focus issue of *Frontiers in Bioengineering and Biotechnology: Nano-Imaging in Translational Cancer Medicine*, we present five articles that discuss a wide breadth of emerging uses of nanoparticles (NPs) for the detection and treatment of cancer. A common theme linking several manuscripts is the use of NPs as a theranostic for the detection and delivery of therapies to melanomas (Guan et al.) and pancreatic cancer (Cai et al.). Taking this one step further into the surgical oncology arena, multidisciplinary clinical perspectives on augmenting the surgical resection of melanoma (Guan et al.) and brain tumors Lam et al. using fluorescence-conjugated tumor-detecting NPs, provide a framework of how nanotechnology is being used to improve surgical outcomes and survival for cancer patients (Figure 1). These publications highlight how far the field of nanomedicine has come since the approval of the liposomal NP formulation of the chemotherapy doxorubicin (Doxil^®^) in 1995 (Barenholz, 2012).

A recent quantitative bibliometric analysis of cancer nanotechnology research published between the years of 2000–2021 mined over 50,000 papers, showing an exponential increase in numbers of papers on nanoparticles published since 2010. Most of these publications originated from institutions located in the United States, China, the United Kingdom, India, and Iran, with an increased ramp up between 2017 and 2021 (Millagaha Gedara et al., 2021). Topics most commonly studied included iron oxide (IONPs) or mesoporous silica NPs, nanotechnology for DNA or RNA packaging, and nanotechnology for the diagnosis and treatment of cancers, in particular, breast, lung, and prostate cancers (Millagaha Gedara et al., 2021). Further analysis of these articles across the top ten cancer types revealed the use of stem cell and xenograft models to study the pharmacodynamic and pharmacokinetics of NP delivery systems and the use of IONPs for preclinical cancer imaging. These trends are nicely recapitulated in this focus issue of *Frontiers*, but the translational theme of the articles also addresses some important limitations and caveats of administering NPs in humans. Kong and Dreaden’s opinion piece on the immunogenicity associated with polyethylene glycol (PEG), commonly used to encapsulate (PEGylate) different types of NPs, thereby shielding them from clearance by the reticuloendothelial system and increasing systemic circulation time, addresses why rigorous safety and quality controls are required before we will see more widespread translation of NPs for in-human use (Kong and Dreaden). The worldwide efforts to control the spread of SARS-CoV-2 (COVID-19) through global administration of a PEGylated liposomal mRNA-based vaccine uncovered a potential underlying hypersensitivity to the PEG component of the vaccine that may hinder its use in future NP formulations across different biomedical applications (de Vrieze and Pfizer, 2021). This adverse hypersensitivity reaction of PEG in the human body potentially puts into question the translatability of the safety profiles and biodistribution data of PEGylated NPs gathered from xenograft models. Case in point, Cai et al. tested the *in vitro* and *in vivo* toxicity profiles of differently PEGylated polymer NPs with fluorescence emission capabilities in the near-infrared (NIR) window in pancreatic cancer cells. Their results clearly demonstrated a wide range of *in vitro* toxicities and *in vivo* accumulation of these differentially PEGylated NPs in subcutaneous flank pancreatic tumors in nude mice (Cai et al.). However, given the recently reported immunogenicity of PEG in humans, one should use an abundance of caution in evaluating the translatability of safety data gathered using immunocompromised animal model systems and addresses the need to design NPs that minimize these adverse events.

Another recurring theme in this issue of *Frontiers* is the use of NPs for the imaging and detection of tumors using NIR fluorescence imaging. These articles are timely as NIR fluorescence-guided surgery (FGS) using the NIR dye indocyanine green (ICG) is increasingly being used for aiding surgical oncologists across multiple specialties in the operating room for visualization of tumor cells that are difficult to discern using conventional white light operating microscopes (Cho et al., 2018; Quan et al., 2020). The review by Faiz et al. outlines the use of IONPs to increase contrast enhancement of high-grade brain tumors on magnetic resonance imaging (MRI) sequences (Faiz et al., 2022), which can then be used in the operating room by neurosurgeons in conjunction with FGS using ICG to better visualize tumor cells (Lam et al.). However, FGS still remains in its infancy and will require rigorous validation before it can be adopted as standard of care practice (Vahrmeijer et al., 2013).

In closing, this focus issue of *Frontiers* showcases emerging and approved nanotechnologies that are poised to help clinical and surgical oncologists offer patients better detection of their tumor burden and delivery of precision cancer therapies. Careful characterization of the toxicity profiles of NPs and adherence to good lab and manufacturing practices for upscale manufacturing will improve the translational value of preclinical phase nanomedicines.
